# Rosiglitazone, an Agonist of PPAR*γ*, Inhibits Non-Small Cell Carcinoma Cell Proliferation In Part through Activation of Tumor Sclerosis Complex-2

**DOI:** 10.1155/2007/29632

**Published:** 2007-06-05

**Authors:** ShouWei Han, Ying Zheng, Jesse Roman

**Affiliations:** ^1^Division of Pulmonary, Allergy, and Critical Care Medicine, Department of Medicine, Emory University School of Medicine, Emory University, Atlanta, GA 30322, USA; ^2^Department of Obstetrics and Gynecology, West China 2nd University Hospital, Sichuan University, Chengdu 610041, China; ^3^Atlanta Veterans Affairs Medical Center, Emory University, Atlanta, GA 30033, USA

## Abstract

PPAR*γ* ligands inhibit the proliferation of non-small cell lung carcinoma (NSCLC) cells in vitro. The mechanisms responsible for this effect remain incompletely elucidated, but PPAR*γ* ligands appear to inhibit the mammalian target of rapamycin (mTOR) pathway. We set out to test the hypothesis that PPAR*γ* ligands activate tuberous sclerosis complex-2 (TSC2), a tumor suppressor gene that inhibits mTOR signaling. We found that the PPAR*γ* ligand rosiglitazone stimulated the phosphorylation of TSC2 at serine-1254, but not threonine-1462. However, an antagonist of PPAR*γ* and PPAR*γ* siRNA did not inhibit these effects. Rosiglitazone also increased the phosphorylation of p38 MAPK, but inhibitors of p38 MAPK and its downstream signal MK2 had no effect on rosiglitazone-induced activation of TSC2. Activation of TSC2 resulted in downregulation of phosphorylated p70S6K, a downstream target of mTOR. A TSC2 siRNA induced p70S6K phosphorylation at baseline and inhibited p70S6K downregulation by rosiglitazone. When compared to a control siRNA in a thymidine incorporation assay, the TSC2 siRNA reduced the growth inhibitory effect of rosiglitazone by fifty percent. These observations suggest that rosiglitazone inhibits NSCLC growth partially through phosphorylation of TSC2 via PPAR*γ*-independent pathways.

## 1. INTRODUCTION

Lung cancer remains the leading cause of cancer-related mortality in the United States, and 30% to 40% of newly diagnosed patients with non-small cell lung cancer (NSCLC) present with regionally advanced and unresectable stage III disease [1]. Despite recent advances in understanding the molecular biology of lung carcinoma and the introduction of multiple new chemotherapeutic agents for its treatment, the poor five-year survival rate of less than 15% has not changed substantially [2]. This justifies the continuous search for agents with therapeutic potential against NSCLC.

Peroxisome proliferator-activated receptors (PPARs; isotypes *α*, *β*/*δ*, *γ*) are ligand-inducible nuclear transcription factors that heterodimerize with retinoid X receptors and bind to PPAR response elements (PPREs) located in the promoter region of PPAR target genes [3]. These lipid-sensitive receptors can be activated in a variable isotype-specific manner by natural fatty acids, leukotrienes, prostaglandins, and some synthetic agonists, including antidiabetic drugs such as rosiglitazone, ciglitazone, and pioglitazone which are specific PPAR*γ* ligands. These drugs are also effective in regulating cell activation, differentiation, proliferation, and/or apoptosis [4, 5]. The role of PPAR*γ*, one PPAR isotype, has been extensively studied thanks to the availability of synthetic PPAR*γ* agonists. The anticancer activity of PPAR*γ* agonists has been examined in a variety of cancers including colon, breast, and prostate [6]. These and related studies support a role for PPAR*γ* as a potential tumor suppressor.

Several studies have implicated PPAR*γ* in lung cancer as well. The expression of PPAR*γ* has been demonstrated in NSCLC and was correlated with tumor histological type and grade [7]. Thus, it has been postulated that PPAR*γ* mRNA levels may serve as a prognostic marker in lung carcinoma in addition to playing important roles in lung carcinogenesis. Activation of PPAR*γ* by troglitazone, ciglitazone, and pioglitazone caused growth inhibition and apoptosis of NSCLC cells [8, 9]. Recently, studies in animal models of tumorigenesis showed that treatment of A549 tumor-bearing SCID mice with troglitazone or pioglitazone inhibited primary tumor growth by 66.7%, and significantly inhibited the number of spontaneous lung metastasis lesions [10]. Together, these observations suggest that PPAR*γ* ligands may serve as potential therapeutic agents in the management of NSCLC, but the mechanisms responsible for these effects remain incompletely elucidated.

We have reported that PPAR*γ* agonists inhibit NSCLC proliferation by inhibiting the mammalian target of rapamycin (mTOR) signaling pathway through PPAR*γ*-dependent and -independent mechanisms [11]. The mTOR subfamily belongs to the phosphatidylinositol 3-kinase (PI3-K)-related kinase family and is partly inhibited by rapamycin, a feature that has facilitated efforts to study its function in eukaryotic cells [12]. mTOR signaling induced by hormones, growth factors, and amino acids regulates the phosphorylation of several proteins including p70 ribosomal protein S6 kinase (p70S6K) and eIF-4E binding protein (4E-BP1), which are key regulators of translation, and are among the most well-characterized targets of mTOR [12].

One of the downregulators of the mTOR pathway is the tumor suppressor protein tuberous sclerosis complex (TSC). TSC is composed of two proteins, TSC1 (also known as hamartin) and TSC2 (known as tuberin), which function to integrate growth factor and cell stress responses [13]. We set out to explore the effects of PPAR*γ* agonists on TSC expression and the contribution of this pathway on inhibition of cell proliferation in NSCLC cells treated with the PPAR*γ* agonist rosiglitazone. We found that PPAR*γ* ligands activate TSC2, which, in turn, inhibits mTOR signaling in NSCLC cells through PPAR*γ*-independent pathways.

## 2. MATERIALS AND METHODS

### 2.1. Culture and chemicals

The human NSCLC cell line H2106 was obtained from the American Type Culture Collection (Manassas, Va, USA) and grown in RPMI-1640 medium supplemented with 10% heat-inactivated FBS, HEPES buffer, 50 IU/mL penicillin/streptomycin, and 1 *μ*g amphotericin (complete medium) as previously described [14]. Polyclonal antibodies specific for TSC2, p38 MAPK, p70S6K, and their respective phosphorylated active forms were purchased from Cell Signaling (Beverly, Mass, USA). GW9662 was purchased from Cayman Chemical Co. (Ann Arbor, Mich, USA). The inhibitor of the mitogen-activated protein kinase-activated protein kinase 2 (MAPKAP kinase 2, MK2), a synthetic 13-residue peptide (KKKALNRQLGVAA) corresponding to the phosphorylation site of HSP27, one of the known substrates of MK2, was purchased from Calbiochem (San Diego, Calif, USA). Rosiglitazone, antibodies against PPAR*γ*, SB239063, and other chemicals were purchased from Sigma Aldrich (St. Louis, Mo, USA) unless otherwise indicated.

### 2.2. Western Blot analysis

Western blotting was performed as previously described [15]. Briefly, protein concentrations were determined by the Bio-Rad protein assay. Equal amounts of protein from whole cell lysates were solubilized in 2x SDS-sample buffer and separated on SDS-8% polyacrylamide gels. Blots were incubated with antibodies raised against TSC2 and phosphorylated TSC2 (1:2000), p38 MAPK and phosphor-p38 MAPK, p70S6K and phosphor-p70S6K (1:1000). The blots were washed and followed by incubation with a secondary goat antibody raised against rabbit IgG conjugated to horseradish peroxidase (1:2000, Cell Signaling, Beverly, Mass, USA). The blots were washed, transferred to freshly made ECL solution (Amersham, Arlington, Ill, USA) for 1 minute, and exposed to X-ray film. In controls, the antibodies were omitted or replaced with a control rabbit IgG.

### 2.3. Treatment with PPAR*γ* and TSC2 small interfering RNA

The PPAR*γ* (Cat number sc-29455) and TSC2 siRNAs (Cat number sc-36762) and the control siRNA (Cat number sc-37007) were purchased from Santa Cruz Biotechnology, Inc. (Santa Cruz, Calif, USA). For the transfection procedure, cells were grown to 50% confluence and PPAR*γ*, TSC2, or control siRNAs were transfected using the Lipofectamine 2000 reagent (Invitrogen, Carlsbad, Calif, USA) according to the manufacturer's instructions. Briefly, oligofectamine reagent was incubated with serum-free medium for 10 minutes. Subsequently, a mixture of siRNA was added. After incubation for 15 minutes at room temperature, the mixture was diluted with medium and added to each well. The final concentration of siRNA in each well was 100 nM. After culturing for 48 hours, cells were washed and resuspended in new culture media in the presence or absence of rosiglitazone for up to 48 hours for Western blot and cell growth assays.

### 2.4. [Methyl-^3^H] thymidine incorporation assay

H2106 NSCLC cells (10^4^ cells/well) were cultured with the selective PPAR*γ* antagonist GW9662 (20 *μ*M) for 1 hour, or transfected with TSC2 siRNA (100 nM) for 48 hours before exposing the cells to rosiglitazone (10 *μ*M) followed by incubation with 1 *μ*Ci/mL [methyl-^3^H] thymidine (Amersham, specific activity 250 Ci/mmol) for up to 48 hours. The medium was removed and the attached cells were washed with 1x PBS. Afterwards, the attached cells were treated with ice-cold 6% trichloroacetic acid (TCA) at 4°C for 20 minutes and washed once with 6% TCA. The cells were then solubilized with 0.1 N NaOH and counted in a liquid scintillation counter in 4 mL of scintillation fluid.

### 2.5. Statistical analysis

All experiments were repeated a minimum of three times. All data collected from Western Blot and [Methyl-^3^H]-thymidine incorporation assays were expressed as means ± SD. The data presented in some figures are from a representative experiment, which was qualitatively similar in the replicate experiments. Statistical significance was determined with Student's *t* test (two-tailed) comparison between two groups of data sets. Asterisks shown in the figures indicate significant differences of experimental groups in comparison with the corresponding control condition (*P* < .05, see figure legends).

## 3. RESULTS

### 3.1. Rosiglitazone stimulates the expression of TSC2 protein

**Figure 1:**
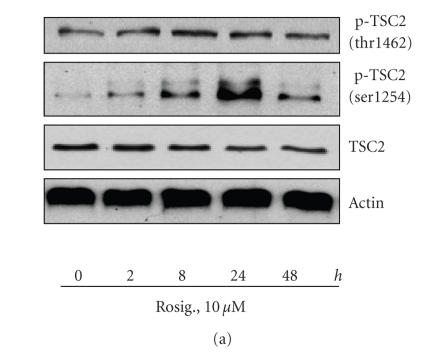

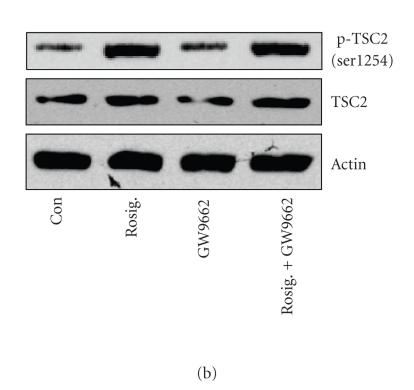

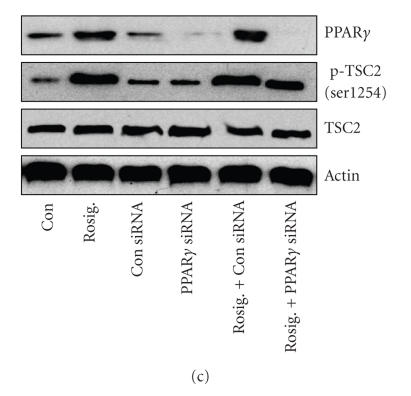
*Rosiglitazone stimulates the activation of TSC2. (a) Time-dependent effect of rosiglitazone on TSC2 phosphorylation*. Cellular protein was isolated from H2106 cells that were cultured with increasing concentrations of rosiglitazone (Rosig.) for 1 hour followed by Western blot analysis with antibodies against total TSC2 and phosphorylated TSC2 (p-TSC2). *(b) Effect of PPAR*γ* antagonists on rosiglitazone-induced TSC2 phosphorylation*. Cellular protein was isolated from H2106 cells cultured for up to 2 hours in the presence or absence of GW9662 (20 *μ*M) before exposure of cells to rosiglitazone (Rosig., 10 *μ*M) for an additional 24 hours, then subjected to Western blot analysis for total TSC2 and phosphorylated TSC2 (p-TSC2). *(c) Effect of PPAR*γ* siRNA on rosiglitazone-induced TSC2 phosphorylation*. H2106 cells were transfected with control or PPAR*γ* siRNA (100 nM each) for 48 hours before exposing the cells to rosiglitazone (Rosig., 10 *μ*M) for up to 24 hours. Afterwards, we performed Western blot analysis for total TSC2 and phosphorylated TSC2 (p-TSC2). Actin served as internal control for normalization purposes (*Con*, indicates untreated control cells).

Since rosiglitazone has been found to regulate the PI3-K/Akt/mTOR/p70S6K signaling pathway, we tested if it also affected TSC2, an upstream regulator of that pathway. H2106 cells treated with rosiglitazone for the indicated period of time showed an increase in the phosphorylation of TSC2 at serine-1254, whereas only a slight increase in phosphorylation was detected on threonine-1462 (Figure 1(a)). Total TSC2 protein levels remained unchanged. PPAR*γ* ligands have been shown to exert their effects through pathways dependent and independent of PPAR*γ*. To test if phosphorylation of TSC2 by rosiglitazone was mediated through activation of PPAR*γ*, cells were pretreated with a selective PPAR*γ* antagonist, GW9662, or PPAR*γ* siRNA before exposing them to rosiglitazone. As depicted in Figures 1(b) and 1(c), the inhibitory effect of rosiglitazone on the phosphorylation of TSC2 was not affected by GW9662 (b) or by PPAR*γ* siRNA (c) suggesting that PPAR*γ*-independent signals mediated this effect. Note that the PPAR*γ* siRNA blocked PPAR*γ* protein production, while the control siRNA had no effect (c).

#### 3.1.1. Rosiglitazone increases the phosphorylation of p38 MAPK, but blockade of p38 MAPK and its downstream signals had no effect on rosiglitazone-induced activation of TSC2

**Figure 2:**
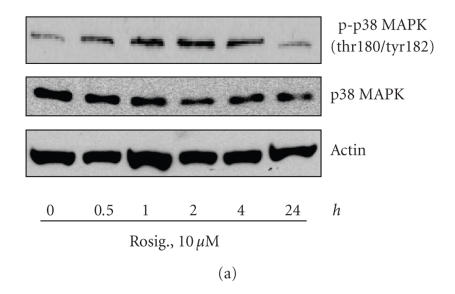

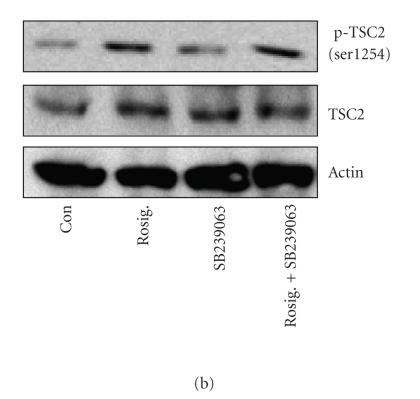

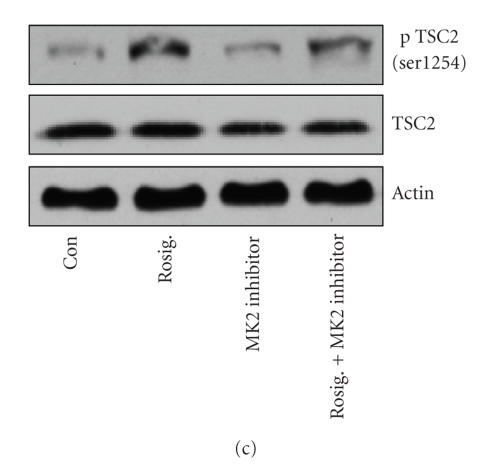
*The role of p38 MAPK signaling cascade in mediating the effect of rosiglitazone on activation of TSC2. (a) Time-dependent effect of rosiglitazone on phosphorylation of p38 MAPK*. Cellular proteins were isolated from H2106 cells treated with rosiglitazone (Rosig., 10 *μ*M) for the indicated time period. Afterwards, Western blot analyses were performed using a polyclonal antibody against phosphor-p38 MAPK (Thr180/Tyr182) and total p38 MAPK. Actin served as internal control for normalization purposes. *(b) Effect of p38 inhibitor on rosiglitazone-induced TSC2 phosphorylation*. Cellular protein was isolated from H2106 cells cultured for up to 2 hours in the presence or absence of SB239063 (10 *μ*M) before exposure of cells to rosiglitazone (Rosig., 10 *μ*M) for an additional 24 hours, then subjected to Western blot analysis for total TSC2 and phosphorylated TSC2 (p-TSC2). Actin served as internal control for normalization purposes (*Con*, indicates untreated control cells). *(c) Effect of MK2 inhibitor on rosiglitazone-induced TSC2 phosphorylation*. Cellular protein was isolated from H2106 cells cultured for up to 2 hours in the presence or absence of MK2 inhibitor (10 *μ*M) before exposure of cells to rosiglitazone (Rosig., 10 *μ*M) for an additional 24 hours, then subjected to Western blot analysis for total TSC2 and phosphorylated TSC2 (p-TSC2). Actin served as internal control for normalization purposes (*Con*, indicates untreated control cells).

PPAR*γ* ligands have been shown to induce the activation of p38 MAPK in different cell systems [16, 17]. Activation of p38 mitogen-activated protein kinase (MAPK) and its downstream kinase MK2 have been associated with the phosphorylation of TSC2 [18]. Similarly, we found that rosiglitazone induced a transient increase in the phosphorylation of p38 MAPK in a time-dependent manner with maximal induction at 2 hours (Figure 2(a)). We next assessed if activation of p38 signals were related to the effect of rosiglitazone on TSC2 activation. As shown in Figures 2(b) and 2(c), SB239063, a selective p38 inhibitor, and KKKALNRQLGVAA, a potent and selective inhibitor of MK2, had no effect on rosiglitazone-induced TSC2 phosphorylation (serine-1254). No effects were noticed with increasing doses of these inhibitors (not shown).

#### 3.1.2. Silencing TSC2 restored the mTOR-related signal and partially blocked the effect of rosiglitazone on cell growth inhibition

**Figure 3:**
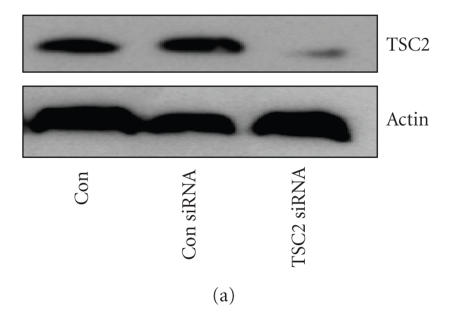

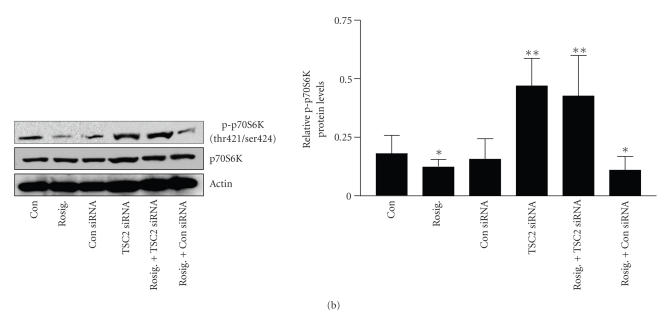
*Silencing TSC2 restored the activity of p70S6K. (a) TSC2 siRNA blocks TSC2 production*. H2106 cells were transfected with control or TSC2 siRNA (100 nM each) for 30 hours. Afterwards, we performed Western blot analysis for TSC2 proteins (Con, indicates untreated cells). *(b) TSC2 siRNA ameliorates the inhibitory effect of rosiglitazone on p70S6K phosphorylation*. H2106 cells were transfected with control or TSC2 siRNA (100 nM each) for 48 hours before exposing the cells to rosiglitazone (Rosig., 10 *μ*M) for up to 24 hours. Afterwards, we performed Western blot analysis for p70S6K proteins (Con, indicates untreated cells). The representative data shown here is obtained from at least three separate experiments. Graphs are densitometry results. (*indicates significant differences as compared to the zero hour or untreated cells (*P* < .05); **indicates significance of combination treatment as compared with rosiglitazone alone (*P* < .05).)

We next examined if upregulation of TSC2 by rosiglitazone was associated with inhibition of mTOR signaling as determined by evaluating the phosphorylation state of p70S6K, a downstream target of mTOR. To determine the exact contribution of TSC2, we tested tumor cells transfected with control and TSC2 siRNAs. As shown in Figure 3(a), the TSC2 siRNA blocked TSC2 protein production, while the control siRNA had no effect. Armed with these tools, we tested the effects of rosiglitazone on p70S6K. As expected, upregulation of TSC2 by rosiglitazone coincided with downregulation of phosphorylated p70S6K (Figure 3(b)). Silencing of TSC2 by siRNA induced phosphorylation of p70S6K at baseline and inhibited p70S6K downregulation in the presence of rosiglitazone demonstrating a direct link between TSC2 induction and inhibition of mTOR signaling (Figure 3(b)).

### 3.2. Rosiglitazone inhibits carcinoma cell proliferation

**Figure 4:**
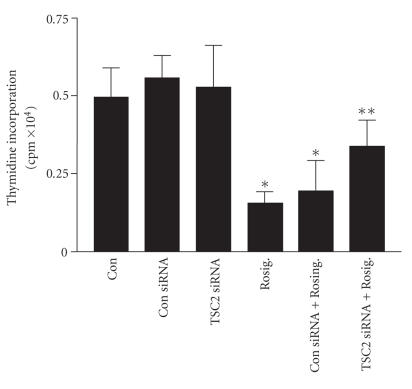
*Silencing TSC2 partly blocked the effect of resiglitazone on cell growth inhibition*. H2106 cells transfected with control or TSC2 siRNA (100 nM each) for 48 hours before exposing the cells to rosiglitazone (Rosig., 10 *μ*M) and incubated with 10 *μ*Ci/mL [methyl-^3^H] thymidine for 48 hours. Afterwards, cell numbers were determined. All data are depicted as means ± SD. (*indicates significant differences as compared to the untreated cells (*P* < .05); **indicates significance of combination treatment as compared with rosiglitazone (Rosig.) alone (*P* < .05).)

We next tested the contribution of TSC2 to NSCLC cell proliferation in the setting of rosiglitazone treatment using an [^3^H] thymidine incorporation assay. As expected, we found that rosiglitazone inhibited NSCLC cell proliferation. This is consistent with our own observations (not shown) and findings by others [10] showing inhibition of tumor growth in vivo in response to rosiglitazone. Interestingly, silencing of TSC2 reduced the growth inhibitory effect of rosiglitazone by approximately 50%, whereas a control siRNA had no effect (Figure 4).

## 4. DISCUSSION

Rosiglitazone, one of the thiazolidinedione derivatives, is the most potent and selective synthetic ligand of PPAR*γ*. It binds to PPAR*γ* with a kD of approximately 40 nM and it is known to have marked adipogenic effects on preadipocyte and mesenchymal stem cells in vitro as well as dramatic antidiabetic effects [19]. However, not all of its cellular effects are mediated via PPAR*γ* [20, 21]. Herein, we show that rosiglitazone increases PPAR*γ* protein expression in NSCLC in a time- and dose-dependent fashion. Note that the concentrations used were consistent with those reported by others. For example, Valentiner et al. found that rosiglitazone inhibited the in vitro growth and viability of human neuroblastoma cell lines in a dose-dependent manner showing considerable effects only at high concentrations (10 *μ*M and 100 *μ*M) [22]. In another study, rosiglitazone inhibited both the proliferation and invasiveness of the human adrenocortical cancer cell line H295R in a dose-dependent manner with maximal effects (about 50% inhibition) noted at 20 *μ*M [23].

We previously demonstrated that rosiglitazone inhibited the activation of the PI3-K/Akt/mTOR signaling pathway in NSCLC cells [11] and, therefore, set out to explore the effects of rosiglitazone on modulators of this pathway. mTOR signaling is induced by hormones, growth factors, and amino acids, and regulates the phosphorylation of several proteins including p70S6K and 4E-BP1, which are key regulators of translation, and are amongst the most well-characterized targets of mTOR [12]. A modulator of the mTOR pathway is the tumor suppressor protein TSC2, which functions to integrate growth factor and cell stress responses [13]. The TSC2 gene is known to be involved in mammalian cell cycle control and its overexpression is thought to exert an antitumor effect on cancer cells [24]. Here, we report that rosiglitazone increased the phosphorylation of TSC2 highlighting the relevance of this tumor suppressor in mediating the effects of rosiglitazone. However, this effect appeared to be independent of PPAR*γ* since the inhibitor of PPAR*γ*, GW9662, and transfection with PPAR*γ* siRNA had no effect on this process.

TSC2 is phosphorylated by multiple kinases, including Akt, RSK1, ERK, CDC2, MK2, and AMPK. Therefore, TSC2 integrates signals from multiple signaling pathways and influences cell growth through regulation of the mTOR pathway [25]. Note that the rosiglitazone-induced phosphorylation of TSC2 occurred at serine-1254, but not at threonine-1462 sites, which are sites different from those phosphorylated by Akt and AMPK [26, 27]. The effect of phosphorylation of TSC2 at serine-1254 site remains unclear [25]. The interaction of TSC2 with 14-3-3 is associated with phosphorylation of serine-1254 and may be independent of Akt. However, TSC2 serine-1254 phosphorylation does not necessarily influence TSC2–14-3-3 interactions [28]. On the contrary, the association between 14-3-3 and TSC2 requires phosphorylation of serine-1210, which is not considered an Akt phosphorylation site [29]. This discrepancy may be due to the different cells studied and the elicitation of mechanisms other than those related to PI3-K, 14-3-3 and p38 pathways. How these multiple phosphorylation events are integrated by TSC2 to regulate cell growth needs to be explored further.

PPAR*γ* ligands have been shown to induce the activation of p38 MAPK in different cell systems [16, 17]. In line with this, we showed that rosiglitazone increased the phosphorylation of p38 MAPK in NSCLC cells. Activation of p38 MAPK and its downstream signal MK2 have been associated with phosphorylation of TSC2 (serine-1210) [18], and the inhibitor of p38 MAPK reduced the phosphorylation of both p38 MAPK and MK2 [30]. However, in the current study, blockade of p38 MAPK and its downstream signal MK2 had no effect on rosiglitazone-induced TSC2 phosphorylation suggesting that the p38 MAPK cascade plays no role in mediating the effect of rosiglitazone on TSC2. The concentrations of these inhibitors were based on other studies which showed significant inhibition of p38 MAPK and its downstream MK2 signaling cascade [30, 31].

TSC1-TSC2 complexes have recently been implicated in cell survival responses. The molecular mechanisms by which TSC2 affects mTOR-related signals remain unclear. We found that knockdown of TSC2 resulted in inhibition of the effect of rosiglitazone on the mTOR downstream target p70S6K suggesting a role for TSC2 in mediating this effect. In cell proliferation assays, we showed that the TSC2 siRNA partially restored NSCLC cell growth in the presence of rosiglitazone, although knockdown of TSC2 alone had no effect on NSCLC cell proliferation. This suggests that TSC2 does not contribute to NSCLC cell proliferation at baseline, but its phosphorylation partially mediates the growth inhibitory effect of rosiglitazone.

Taken together, our results demonstrate that rosiglitazone inhibits NSCLC cell growth in part through activation of TSC2 with the consequent suppression of mTOR signaling. This effect appeared to be independent of PPAR*γ* and p38 MAPK signaling pathways. This work complements our previous work demonstrating partial inhibition of the mTOR pathway by rosiglitazone through downregulation of Akt and induction of PTEN via PPAR*γ*-dependent pathways [11]. Together, the activation of rosiglitazone-induced PPAR*γ*-dependent and -independent pathways results in inhibition of NSCLC growth. These observations are justifying further work testing the use of rosiglitazone (and perhaps other PPAR*γ* ligands) as potential coadjuvants in the treatment of NSCLC in humans.

## ABBREVIATIONS

NSCLC: Non-small cell lung carcinoma
PPAR*γ*: Peroxisome proliferator-activated receptor gamma
PPRE: PPAR response element
PI3-K: Phosphatidylinositol 3-kinase
p38 MAPK: p38 mitogen-activated protein kinase
MK2: MAPKAP kinase 2
mTOR: Mammalian target of rapamycin
p70S6K: p70 ribosomal S6 kinase
TSC2: Tuberous sclerosis complex-2
4E-BP1: Eukaryotic initiation factor 4E-binding protein 1
siRNA: Small RNA interference
